# 3D Gel Map of *Arabidopsis* Complex I

**DOI:** 10.3389/fpls.2013.00153

**Published:** 2013-06-04

**Authors:** Katrin Peters, Katharina Belt, Hans-Peter Braun

**Affiliations:** Institute for Plant Genetics, Faculty of Natural Sciences, Leibniz Universität Hannover, Hannover, Germany

**Keywords:** mitochondria, OXPHOS system, respiratory chain, NADH dehydrogenase, blue-native, BN/SDS/SDS-PAGE, *Arabidopsis thaliana*

## Abstract

Complex I has a unique structure in plants and includes extra subunits. Here, we present a novel study to define its protein constituents. Mitochondria were isolated from *Arabidopsis thaliana* cell cultures, leaves, and roots. Subunits of complex I were resolved by 3D blue-native (BN)/SDS/SDS-PAGE and identified by mass spectrometry. Overall, 55 distinct proteins were found, seven of which occur in pairs of isoforms. We present evidence that *Arabidopsis* complex I consists of 49 distinct types of subunits, 40 of which represent homologs of bovine complex I. The nine other subunits represent special proteins absent in the animal linage of eukaryotes, most prominently a group of subunits related to bacterial gamma-type carbonic anhydrases. A GelMap http://www.gelmap.de/arabidopsis-3d-complex-i/ is presented for promoting future complex I research in *Arabidopsis thaliana*.

## Introduction

The NADH dehydrogenase complex (complex I) of the Oxidative Phosphorylation (OXPHOS) system is present in the cytoplasmic membrane of aerobic bacteria and the inner mitochondrial membrane of eukaryotes. It is composed of two elongated arms: the “membrane arm,” and the so-called “peripheral arm” which protrudes into the cytoplasm of the bacterial cell or the matrix of mitochondria (reviewed in Friedrich and Böttcher, 2004; Brandt, 2006; Vogel et al., 2007; Remacle et al., 2008; Zickermann et al., 2008, 2009; Lazarou et al., 2009). The two arms form an L-like structure as originally revealed by electron microscopy (Hofhaus et al., 1991). Very recently, the structure of the entire bacterial enzyme complex has been resolved by X-ray crystallography (Baradaran et al., 2013). Complex I represents a NADH:ubiquinone oxidoreductase. Electron transfer entirely takes place within the peripheral arm and involves an electron transfer chain composed of seven FeS clusters (Hinchliffe and Sazanov, 2005). Quinone reduction takes place at the interface between the two arms and was proposed to induce an electrostatical chain reaction throughout the membrane arm which drives proton translocation across the bacterial or mitochondrial membrane (Baradaran et al., 2013).

Complex I is by far the largest complex of the OXPHOS system. In its simplest form, the bacterial complex consists of 14 subunits (seven subunits per arm) and has a molecular mass of about 500 kDa. However, in eukaryotes, complex I is much larger and consists of more than 40 subunits. Bovine complex I, which extensively was investigated with respect to its subunit composition, consists of 44 subunits, 16 of which are localized in the peripheral and 28 in the membrane arm (Carroll et al., 2006; Balsa et al., 2012). Complex I composition is remarkably conserved in different eukaryotic lineages (Cardol, 2011). However, some lineage-specific complex I subunits occur (Cardol, 2011).

Additional subunits were especially described for plants. Using electron microscopy, complex I of plants was shown to have a very unique shape (Dudkina et al., 2005; Sunderhaus et al., 2006; Peters et al., 2008; Bultema et al., 2009). It has an extra spherical domain which is attached to the membrane arm at a central position and, like the peripheral arm, protrudes into the mitochondrial matrix. It was shown to include extra subunits which resemble gamma-type carbonic anhydrases (Perales et al., 2005; Sunderhaus et al., 2006). In *Arabidopsis*, three carbonic anhydrase subunits form part of complex I (termed CA1, CA2, and CA3) and additionally two more derived “carbonic anhydrase-like” proteins (CAL1 and CAL2). Proteomic studies were initiated to systematically characterize complex I subunits in plants (Heazlewood et al., 2003; Cardol et al., 2004; Sunderhaus et al., 2006; Meyer et al., 2008; Klodmann et al., 2010, 2011; Klodmann and Braun, 2011; Li et al., 2013). These projects led to the identification of several proteins homologous to subunits of bovine complex I and some additional subunits specifically occurring in plants. However, resulting protein sets slightly differ between the presented studies (reviewed in Meyer, 2012).

Here, we present a new study to thoroughly characterize complex I subunits in the model plant *Arabidopsis thaliana*. Our study is based on a 3D gel-electrophoretic approach introduced by Meyer et al. (2008). Using mass spectrometry (MS), 55 complex I proteins were identified, seven of which occur in pairs of isoforms. We present evidence that complex I of *Arabidopsis* includes at least 49 types of proteins, 40 of which represent homologs of bovine complex I and 9 of which are special to plants. A 3D GelMap is presented at http://www.gelmap.de/Arabidopsis-3D-complex-I to facilitate future complex I research in *Arabidopsis*.

## Materials and Methods

### Plant material

A cell culture of *Arabidopsis thaliana* (Col-0) was established as described by May and Leaver (1993). Callus was maintained as suspension culture according to Sunderhaus et al. (2006). Leaves were harvested from 3 weeks old *Arabidopsis thaliana* (Col-0) plants grown in soil at long day conditions (16 h light, 8 h dark) at 22 °C during the day and 20 °C at night. *Arabidopsis* roots were cultured in liquid medium as described by Lee et al. (2011). For this approach, 50–100 seeds of *Arabidopsis thaliana* Col-0 were surface-sterilized in 70% ethanol for 5 min followed by 5 min incubation in 5% bleach/0.1% Tween 20. Seeds were then washed five times in sterilized water. Length of the single washing steps was increased from 10 s to finally 5 min. All incubation steps took place in a rotary shaker. After the final washing step an appropriate volume of 0.15% agarose solution was added to the seeds. The seeds immediately were carefully dispensed on a stainless steel wire mesh platform which is part of the hydroponic culture system adapted from Schlesier et al. (2003). Conditions for hydroponic culture were according to the protocol of Schlesier et al. (2003). *Arabidopsis* plants were grown under 16/8 h light/dark period with light intensity 100–125 μmol m^−2^ s^−1^ at 22 °C. Liquid medium was replaced with freshly made liquid medium after 2 weeks. After 4 weeks the roots were harvested, pre-washed in root culture medium [0.38% (w/v) Gamborg’s B5 salt with vitamins, 3% sucrose, pH 5.8] and transferred into Erlenmeyer flasks containing 50 ml root culture medium. The root culture was kept at 22 °C in the dark under constant agitation at 100 rpm (Lee et al., 2011). It was maintained by transferring small amounts of roots into a new culture flask containing freshly prepared sterilized root culture medium every 3 weeks.

### Isolation of mitochondria

Mitochondria from cell culture were isolated as described by Werhahn et al. (2001). Isolation of mitochondria from green leaves and roots was performed according to the protocol of Keech et al. (2005).

### 3D BN/SDS/SDS-PAGE

One-dimensional blue-native PAGE (1D BN-PAGE) was performed according to Wittig et al. (2006). Mitochondrial membranes were solubilized by digitonin at a concentration of 5 g/g mitochondrial protein (Eubel et al., 2003). The two further gel dimensions represented a 2D SDS/SDS-PAGE as originally suggested by Rais et al. (2004). Combining 1D BN-PAGE and 2D SDS/SDS-PAGE was carried out according to Meyer et al. (2008). For this approach, bands corresponding to complex I were excised from the blue-native (BN) gel. Three bands of complex I were used to build a stack on top of a SDS gel (10% polyacrylamide). Electrophoresis was carried out in the presence of 6 M urea. After end of the electrophoretic run, the lane was cut out from the second gel dimension and incubated in acidic solution (Meyer et al., 2008). The gel strip then was horizontally transferred on top of a third dimension SDS gel (16% polyacrylamide) and gel electrophoresis was carried out in the absence of urea.

### Gel staining procedures

Polyacrylamide gels were stained with Coomassie Brilliant Blue G250 according to the protocol of Neuhoff et al. (1988, 1990).

### Protein identification by mass spectrometry

Tryptic digestion of proteins and identification of proteins by MS were performed as described by Klodmann et al. (2010). Procedures were based on peptide separation using the EASY-nLC System (Proxeon; Thermo Scientific, Bremen, Germany) and coupled MS analyses using the MicrOTOF-Q II mass spectrometer (Bruker Bremen, Germany). MS data evaluation was carried out using ProteinScape2.1 software (Bruker, Bremen, Germany), the Mascot search engine (Matrix Science, London, UK), and (1) the *Arabidopsis* protein database^1^ as well as (2) an updated version of the complex I database used by Klodmann et al. (2010). The latter database is also based on the TAIR protein database (release 10) and includes additionally proteins known to co-migrate with complex I on Blue-native gels (like prohibitins). The following Mascot search parameters were used: enzyme, trypsin/P (up to one missed cleavage allowed); global modification, carbamidomethylation (C), variable modifications, acetyl (N), oxidation (M); precursor ion mass tolerance, 15 ppm; fragment ion mass tolerance, 0.05 Da; peptide charge, 1+, 2+, and 3+; instrument type, electrospray ionization quadrupole time of flight. Minimum ion score was 15, minimum peptide length was four amino acids, significance threshold was set to 0.05 and protein and peptide assessments were carried out if the Mascot Score was greater than 30 for proteins and 20 for peptides.

### Image processing and database generation using GelMap

Coomassie-blue stained 3D BN/SDS/SDS gels of complex I were scanned using the Image Scanner III (GE Healthcare). Spot coordinates were generated using Microsoft Office Paint. The gel image and a file containing all relevant MS data including the spot coordinates were exported into the GelMap software package available at www.gelmap.de following the instructions given on the website and in Senkler and Braun (2012).

## Results and Discussion

### Separation of complex I subunits by 3D gel electrophoresis

**Figure 1:**
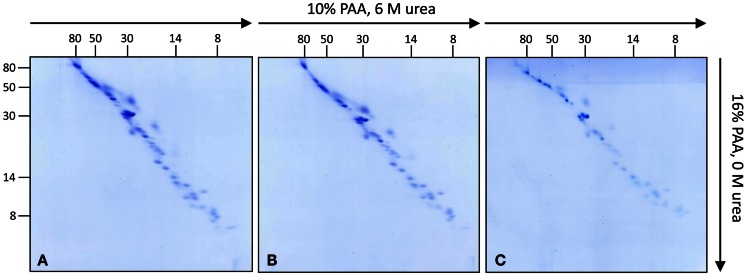
**Investigation of complex I subunits from different tissues of *Arabidopsis thaliana* by 3D BN/SDS/SDS-PAGE**. Total mitochondrial protein from cell culture, leaves, and roots (1200 μg each) was resolved by BN-PAGE in a first dimension. Complex I was cut out from the BN gel and used for second gel dimensions [SDS-PAGE within a 10% polyacrylamide (PAA) gel in the presence of 6 M urea]. Lanes from the second dimension gels were again cut out and transferred horizontally onto third gel dimensions (SDS-PAGE within a 16% PAA gel in the absence of urea). Gels were stained with Coomassie colloidal. **(A)** Complex I of cell cultures, **(B)** of leaves, **(C)** of roots. Molecular masses (in kilodaltons) are given to the left and on the top of the gels.

To further investigate the subunit composition of *Arabidopsis* complex I, isolated mitochondria from leaves, roots, and cell cultures were analyzed by 3D BN/SDS/SDS-PAGE according to Meyer et al. (2008) (Figure S1 in Supplementary Material). In the first gel dimension intact mitochondrial protein complexes are resolved by BN-PAGE. Bands representing mitochondrial complex I are cut out from the gel and staples of up to three bands are transferred onto the 2D SDS/SDS-PAGE system as published by Rais et al. (2004). The latter electrophoresis system combines the advantages of high resolution SDS-PAGE with differential resolution of hydrophilic versus hydrophobic proteins. The first SDS gel dimension contains 10% polyacrylamide (PAA) plus 6 M urea while the second SDS gel dimension contains no urea and has a PAA concentration of 16%. On the resulting SDS/SDS gels proteins are dispersed around a diagonal line. This variation in electrophoretic mobility is presumably caused by an altered interaction between SDS and proteins in the presence or absence of urea (Rais et al., 2004). Furthermore, highly hydrophobic proteins show a differential electrophoretic mobility in gels with varying PAA concentrations. In low PAA gels, hydrophobic proteins run slightly faster than hydrophilic ones and in high PPA gels the other way round. On the 2D gel system suggested by Rais et al. (2004) hydrophobic proteins run above the diagonal line. Since complex I likewise includes highly hydrophobic and hydrophilic subunits this gel system nicely allows to investigate its composition (Rais et al., 2004; Meyer et al., 2008; Angerer et al., 2011; Dröse et al., 2011). Upon optimization of protocols, 3D BN/SDS/SDS-PAGE of complex I from *Arabidopsis* cell culture, leaves, and roots allowed to visualize 52 protein spots per fraction based on Coomassie-staining (Figure 1; Figure S2 in Supplementary Material). Variation in subunit composition between the three *Arabidopsis* tissues was not observed.

### Analysis of complex I subunits

**Figure 2:**
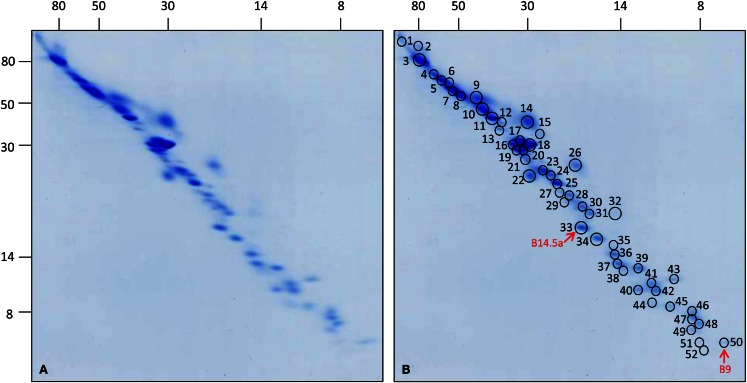
**3D map of complex I from *Arabidopsis thaliana* cell culture**. Total mitochondrial protein (1200 μg each) was resolved by 3D BN/SDS/SDS-PAGE. **(A)** Coomassie-stained gel, **(B)** same gel as in **(A)** indicating protein spots which have been analyzed by mass spectrometry. Numbers correspond to those given in Table 1. Red arrows indicate the newly identified subunits B14.5a and B9. Molecular masses (in kilodaltons) are given to the left and on the top of the gels.

All 52 protein spots of complex I from cell culture and selected subunits of complex I from leaves and roots were analyzed by ESI MS/MS (Figure 2; Table 1; Figure S2 and Tables in Supplementary Material). Overall, 55 distinct proteins were identified. Analyses of two spots in the low-molecular-mass range did not allow identifying any proteins (spots 51 and 52 on Figure 2). Due to spot overlappings, some proteins were detected in more than one spot. The main locations of all proteins (here: highest Mascot score) as well as their secondary locations on the gel are given in Table 1. Overall, 7 out of the 55 subunits of *Arabidopsis* complex I occur in pairs of isoforms. This reduces the number of distinct types of subunits detected in our complex I fraction to 48. The subunit ND4L was not detected by MS in our or any previous investigation on *Arabidopsis* complex I which is most likely due to its extreme hydrophobicity (gravy score + 0.976). Systematic analysis of the subunit composition of complex I in the model organism *Yarrowia lipolytica* also did not led to the identification of this subunit (Abdrakhmanova et al., 2004). ND4L belongs to the “core” set of subunits present in all complex I particles. Its gene is localized on the mitochondrial genome in *Arabidopsis*, transcribed and edited (Giegé and Brennicke, 1999). We speculate that ND4L is represented by spots 51 or 52 in the 7 kDa range of our 3D gel, both of which could not be identified (Figure 2; Figure S3 in Supplementary Material). ND4L has a calculated mass of 10.9 kDa but is very hydrophobic and therefore should run at ∼7 kDa upon SDS-PAGE. We conclude that *Arabidopsis* complex I consists of at least 49 subunits, 48 of which were detected by our analyses, seven of which occur in pairs of isoforms.

**Table 1:** **Complex I subunits in *Arabidopsis thaliana***.

Subunit^1^		Accession^2^	Spot^3^		Organ^4^

	
Plant subunit	Bovine homolog		Main spot	Further spots	
***Membrane arm***					
15 kDa-1	15 kDa	At3g62790	41		c
15 kDa-2	15 kDa	At2g47690	41		c
AGGG	AGGG	At1g76200	47		c
ASHI	ASHI	At5g47570	44		c
B9	B9	At2g46540	50		c
B12-1	B12	At1g14450	45		c, l
B12-2	B12	At2g02510	45		c, l
B14	B14	At3g12260	36		c
B14.5b	B14.5b	At4g20150	47		c, l
B14.7	B14.7	At2g42210	33		c, l
B15	B15	At2g31490	46		c, l
B16.6-1	B16.6	At1g04630	34		c, l
B16.6-2	B16.6	At2g33220	34		c
B18	B18	At2g02050	38	37	c, l, r
B22	B22	At4g34700	35		c
ESSS-1	ESSS	At2g42310	42		c
ESSS-2	ESSS	At3g57785	42		c
KFYI	KFYI	At4g00585	41	32	c
MNLL	MNLL	At4g16450	40		c, l, r
MWFE	MWFE	At3g08610	49		c, l
ND1	ND1	AtMg00516/AtMg01120/AtMg01275^5^	26		c, l
ND2	ND2	AtMg00285/AtMg01320^5^	14		c, l, r
ND3	ND3	AtMg00990	43		c
ND4	ND4	AtMg00580	14		c
ND4L	ND4L	AtMg00650	–		–
ND5	ND5	AtMg00060/AtMg00513/AtMg00665^5^	9	2	c, l
ND6	ND6	AtMg00270	15		c
PDSW-2	PDSW	At1g49140	36		c, l, r
PDSW-1	PDSW	At3g18410	36		c, l, r
PGIV-1	PGIV	At3g06310	37		c
PGIV-2	PGIV	At5g18800	38	37	c
GLDH	–	At3g47930	4		c
P1	–	At1g67350	39		c, l
P2	–	At2g27730	37		c, l, r
At1g18320	–	At1g18320	29		c
***Carbonic anhydrase domain (membrane arm)***					
CA1	–	At1g19580	16	17, 18, 19, 20	c, l
CA2	–	At1g47260	16	13, 17, 18, 19, 20	c, l
CA3	–	At5g66510	21	19, 20	c, l
CAL1	–	At5g63510	23		c, l
CAL2	–	At3g48680	23	22, 24	c, l, r
***Peripheral arm***					
13 kDa	13 kDa	At3g03070	44		c
18 kDa	18 kDa	At5g67590	33		c, l
24 kDa	24 kDa	At4g02580	21		c, l, r
39 kDa	39 kDa	At2g20360	11	12	c, l, r
51 kDa	51 kDa	At5g08530	8	5, 6, 7, 9	c, l
75 kDa	75 kDa	At5g37510	3	1, 2, 4	c, l
B8	B8	At5g47890	42	41	c, l
B13	B13	At5g52840	28	27, 29	c, l, r
B14.5a	B14.5a	At5g08060	33		c
B17.2	B17.2	At3g03100	31		c
ND7	49 kDa	AtMg00510	10		c, l
ND9	30 kDa	AtMg00070	25		c, l, r
PSST	PSST	At5g11770	30		c, l, r
SGDH	SGDH	At1g67785	48		c, l
TYKY-1	TYKY	At1g79010	22		c, l
TYKY-2	TYKY	At1g16700	22		c, l

*^1^Subunits of complex I from Arabidopsis were named according to their homologs in bovine complex I (40 homologous subunits). Exceptions: Arabidopsis homologs to the 30 and 49 kDa subunits of bovine complex I are designated ND7 and ND9 because the corresponding proteins are encoded by the mitochondrial genome in plants. Seven subunits occur in pairs of isoforms in Arabidopsis. The names of these proteins were extended by “−1” and “−2.” Arabidopsis complex I includes nine additional subunits absent in bovine complex I. These proteins are named in accordance to the literature: CA1, CA2, CA3, CAL1, CAL2, GLDH (L-galactone 1-4 lactone dehydrogenase), P1, P2, and At1g18320*.

*^2^Accession numbers as given by TAIR http://www.arabidopsis.org*.

*^3^Spot number in accordance with Figure 2*.

*^4^Organ/culture in which the subunit was identified; c, cell culture; l, leaf; r, root*.

*^5^Two to three accession numbers are given for the ND1, ND2, and ND5 proteins because they are encoded by a corresponding number of gene fragments on the mitochondrial genome in Arabidopsis. Transcripts encoding the complete proteins are generated by trans-splicing (Knoop et al., 1991; Knoop and Brennicke, 1993; Lippok et al., 1996)*.

For a limited number of subunits, MS analysis also was carried out for the *Arabidopsis* leaves and roots fractions (Table 1; Table S1 in Supplementary Material). Identifications confirm the results obtained for the *Arabidopsis* cell culture. However, in some cases the main locations of corresponding subunits slightly vary between the fractions. It cannot be excluded that these differences are caused by minor gel to gel variations which in some cases made it difficult to precisely assign spots between different fractions. Possible variations in complex I subunit composition between different *Arabidopsis* fractions should be further addressed by future studies.

Based on previous topological investigations for *Arabidopsis* and other model organisms (Carroll, 2003; Hunte et al., 2010; Klodmann et al., 2010; Angerer et al., 2011; Cardol, 2011; Dröse et al., 2011), all 49 subunits can be assigned to the membrane or the peripheral arm of complex I. The peripheral arm consists of 15 subunits, the membrane arm of 34 subunits (Table 1). Five subunits of the membrane arm form part of the so-called carbonic anhydrase (CA/CAL) domain, which is absent in mitochondria of opisthokonts (animals and fungi; Gawryluk and Gray, 2010; Cardol, 2011). Of the 49 subunits, 40 represent homologs of subunits present in bovine complex I (Table 1). Two of these proteins (subunits B14.5a and B9) were identified for the first time in *Arabidopsis* but previously predicted to form part of complex I by genome analyses (Cardol, 2011). The high number of homologs in bovine and *Arabidopsis* complex I underlines the remarkable conservation of this protein complex in Eukaryotes (Cardol, 2011). Bovine complex I consists of 44 subunits (Carroll et al., 2006; Balsa et al., 2012), only four of which were not found in *Arabidopsis* (10 kDa, 42 kDa, SDAP, and B17 subunits; Meyer, 2012). On the contrary, *Arabidopsis* complex I includes nine subunits absent in the bovine complex (for summary, see Figure S4 in Supplementary Material).

Of the nine extra subunits in plants, five represent members of the CA/CAL family. Since deletion of single CA or CAL genes does not cause complete loss of intact complex I (Perales et al., 2005; Sunderhaus et al., 2006; Meyer et al., 2011; Wang et al., 2012a) it cannot be excluded that they present isoforms which alternatively are present in complex I particles. However, deletion of the ca2 gene leads to highly reduced levels of complex I (Perales et al., 2005) indicating that CA2 cannot easily be replaced by CA1 or CA3. Sequence identity between CA1, CA2, and CA3 is in the range of 75%. In contrast, sequences of the CAL1 and CAL2 subunits of *Arabidopsis* are very similar (90% sequence identity), possibly indicating that these proteins represent isoforms. Indeed, deletion of the cal1 or cal2 gene in *Arabidopsis* does not visibly affect *Arabidopsis* development but the double mutant is not viable (Wang et al., 2012a). Considering the size of the CA/CAL domain upon single particle EM of *Arabidopsis* complex I it was concluded that it consists of at least three copies of CA/CAL proteins (Sunderhaus et al., 2006). Further experiments have to be carried out in order to clarify the number of CA/CAL subunits per individual complex I particles.

The plant-specific GLDH subunit binds to three complex I assembly intermediates of 420, 480, and 850 kDa (Schertl et al., 2012) but so far was not detected in preparations of intact complex I. Our data point to the possibility that GLDH also binds to the intact complex. However, it cannot be excluded that the 1000 kDa complex I band excised from the BN gel also included small amounts of the band representing the 850 kDa subcomplex. Three further plant-specific subunits were detected on our 3D gels: P1, P2, and a protein encoded by At1g18320. The P1 and P2 proteins were consistently detected in complex I fractions from plants (Meyer, 2012). Both form part of the membrane arm (Sunderhaus et al., 2006). The At1g18320 protein was previously found to co-migrate with complex I on a BN/SDS gel (Klodmann et al., 2011). However, its status representing an integral complex I subunit in *Arabidopsis* should be further investigated.

### Further complex I subunits in plants?

In previous investigations based on BN-PAGE six additional complex I proteins were identified in *Arabidopsis* (summarized in Meyer, 2012): At5g14105 (Klodmann et al., 2010; Klodmann and Braun, 2011), At1g68680 (Meyer et al., 2008), At1g72170, At3g10110 and At2g28430 (Klodmann et al., 2011), and At1g72750 (Wang et al., 2012b) (Table 2). However, detection of these proteins is not consistent. It currently cannot be excluded that these proteins co-migrate with complex I on blue-native gels but form part of separate complexes. Interestingly, some of these proteins are known components of the pre-protein translocase of the inner mitochondrial membrane, the TIM complex (At1g72750 and At3g10110; the latter protein represents an isoform of At1g18320 which was identified in the course of our current study; Table 1). It recently has been suggested that complex I and the TIM complex are physically linked in plant mitochondria (Murcha et al., 2012).

**Table 2:** **Candidates of additional complex I subunits in *Arabidopsis thaliana***.

Accession	Evidence	Remark
At5g14105	Klodmann et al. (2010), Klodmann and Braun (2011)	Subunit P3^1^
At1g68680	Meyer et al. (2008)	
At3g10110	Klodmann et al. (2011)	Similar to TIM22
At1g72170	Klodmann et al. (2011)	
At2g28430	Klodmann et al. (2011)	
At1g72750	Wang et al. (2012b)	Similar to TIM23

*^1^At5g14105 was suggested to be named P3 in Meyer (2012)*.

### 3D reference map of complex I

To facilitate identifying complex I subunits upon 3D BN/SDS/SDS-PAGE, a GelMap was generated for the MS dataset of the gel presented in Figure 2. GelMap is a software tool for the building and presentation of proteome reference maps (www.gelmap.de; Senkler and Braun, 2012). In contrast to alternative software packages, it allows assignment of multiple proteins per protein spot and at the same time functional annotation of all proteins. By clicking onto protein spots, widespread information is offered. Several GelMaps on *Arabidopsis* mitochondria are presented at the GelMap homepage, including a map on SDS-induced complex I subcomplexes^2^.

**Figure 3:**
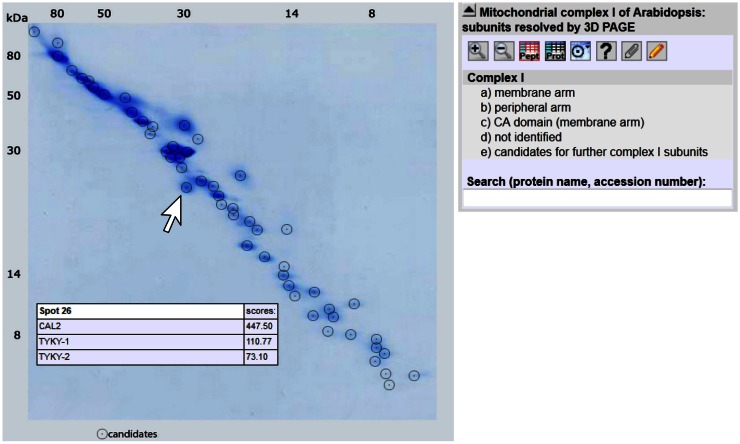
**GelMap of complex I as resolved by 3D BN/SDS/SDS-PAGE (http://www.gelmap.de/arabidopsis-3d-complex-i/)**. Upon hovering with the cursor over a spot, a tooltip including information on all included proteins is opened. In the example given on the figure, the indicated spot includes the CAL2 protein and two isoforms of the TYKY subunit. Upon clicking into the spot the protein names are converted into stable links which can be used to obtain further information. Protein information also can be obtained by clicking into the menu given to the right or by entering protein names or accessions into the search field below the menu.

For the 3D GelMap of *Arabidopsis* complex I, the 55 identified proteins are grouped into functional categories according to their localization within the peripheral arm, the membrane arm, or the carbonic anhydrase domain attached to the membrane arm (Figure 3; http://www.gelmap.de/arabidopsis-3d-complex-i/). Furthermore, the six candidates for additional complex I subunits are given in another category. The proteins of the latter category are linked to an “extra” spot below the gel. By clicking onto any protein spot on the map, all included proteins are displayed. Proteins are sorted according to their MASCOT scores. Upon clicking onto an individual protein, a tooltip opens which includes additional information. Extensive further information on each protein is offered by links to several external databases. The new GelMap is intended to be a helpful tool for future complex I research in *Arabidopsis*.

## Conflict of Interest Statement

The authors declare that the research was conducted in the absence of any commercial or financial relationships that could be construed as a potential conflict of interest.

## Supplementary Material

The Supplementary Material for this article can be found online at: http://www.frontiersin.org/Plant_Proteomics/10.3389/fpls.2013.00153/abstract

Supplementary Figure S1 **Principle of 3D BN/SDS/SDS-PAGE**.

Supplementary Figure S2 **Replicates of 3D BN/SDS/SDS gels for complex I from cell cultures of *Arabidopsis***.

Supplementary Figure S3 **Regions on 3D BN/SDS/SDS gels showing the smallest complex I subunits**. Gels were Coomassie stained (left, middle) or silver stained (right). The three smallest proteins (corresponding to spots 50, 51, and 52 on Figure 2) only become clearly visible upon silver staining. Spot 50 represents the B9 subunit. Spot 51 might represent subunit ND4L. Spot 52 could not be identified.

Supplementary Figure S4 **Species specific complex I subunits in *B. taurus* and *A. thaliana***.

Supplementary Figure S5 **Identity of complex I subunits of *Arabidopsis* upon analysis by 3D BN/SDS/SDS PAGE**.

Supplementary Table S1 **Protein table of the GelMap (http://www.gelmap.de/arabidopsis-3d-complex-i/)**.

Supplementary Table S2 **Protein table of complex I subunits in leaves of *Arabidopsis thaliana***.

Supplementary Table S3 **Protein table of complex I subunits in roots of *Arabidopsis thaliana***.

## Footnotes

^1^www.Arabidopsis.org; release TAIR 10

^2^http://www.gelmap.de/177
