# Supplementary material for: 3D Gel Map of Arabidopsis Complex I
Source: Front Plant Sci. 2013 Jun 4;4:153. doi: 10.3389/fpls.2013.00153 (PMC3671202; doi:10.3389/fpls.2013.00153)
Supplement: Supplementary Figure S1 — Principle of 3D BN/SDS/SDS-PAGE. [file 50654_Braun_Presentation1.PPTX]

## Slide 1
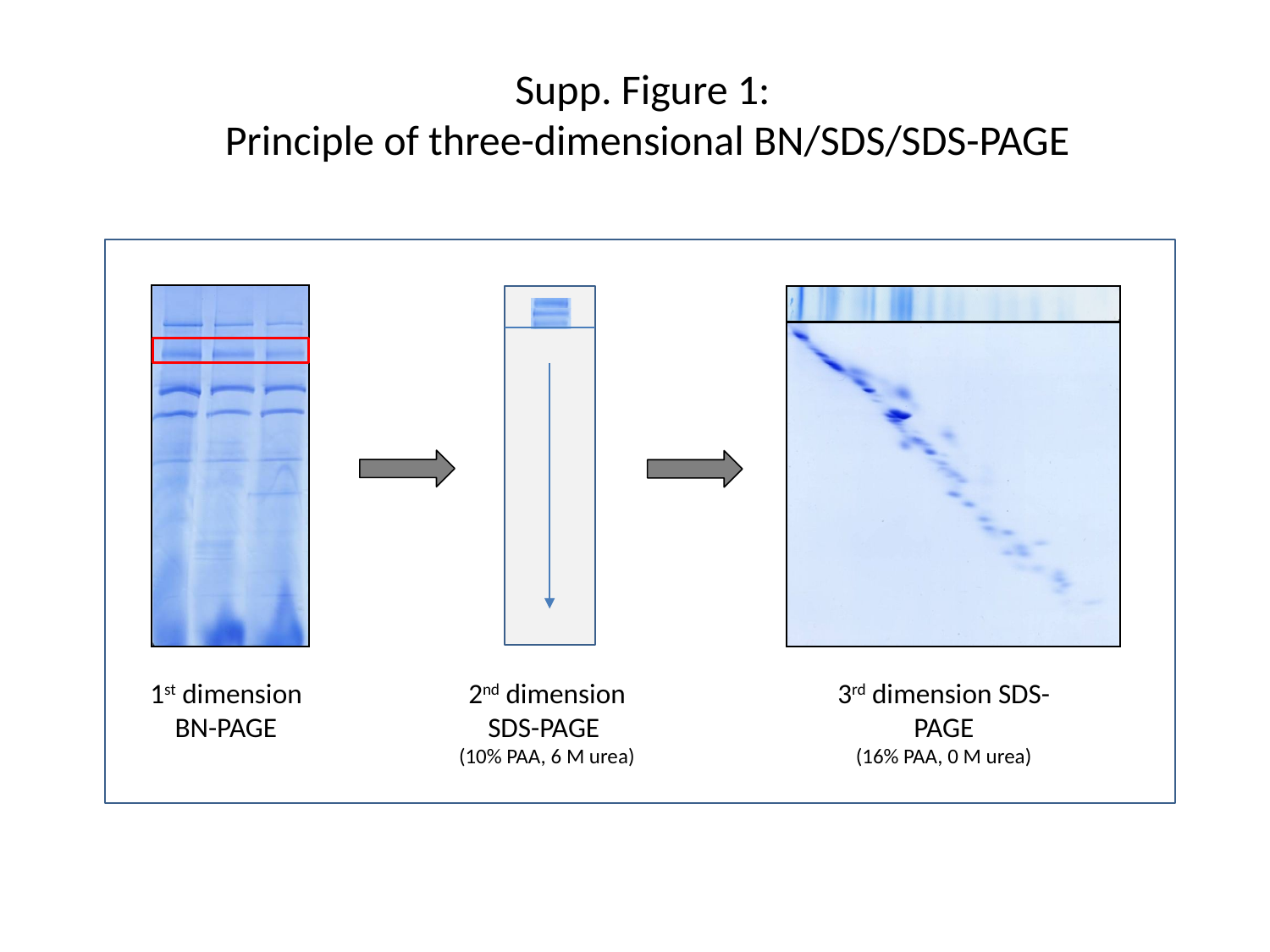

Supp. Figure 1:
Principle of three-dimensional BN/SDS/SDS-PAGE
1st dimension
BN-PAGE
2nd dimension SDS-PAGE
(10% PAA, 6 M urea)
3rd dimension SDS-PAGE
(16% PAA, 0 M urea)

## Slide 2
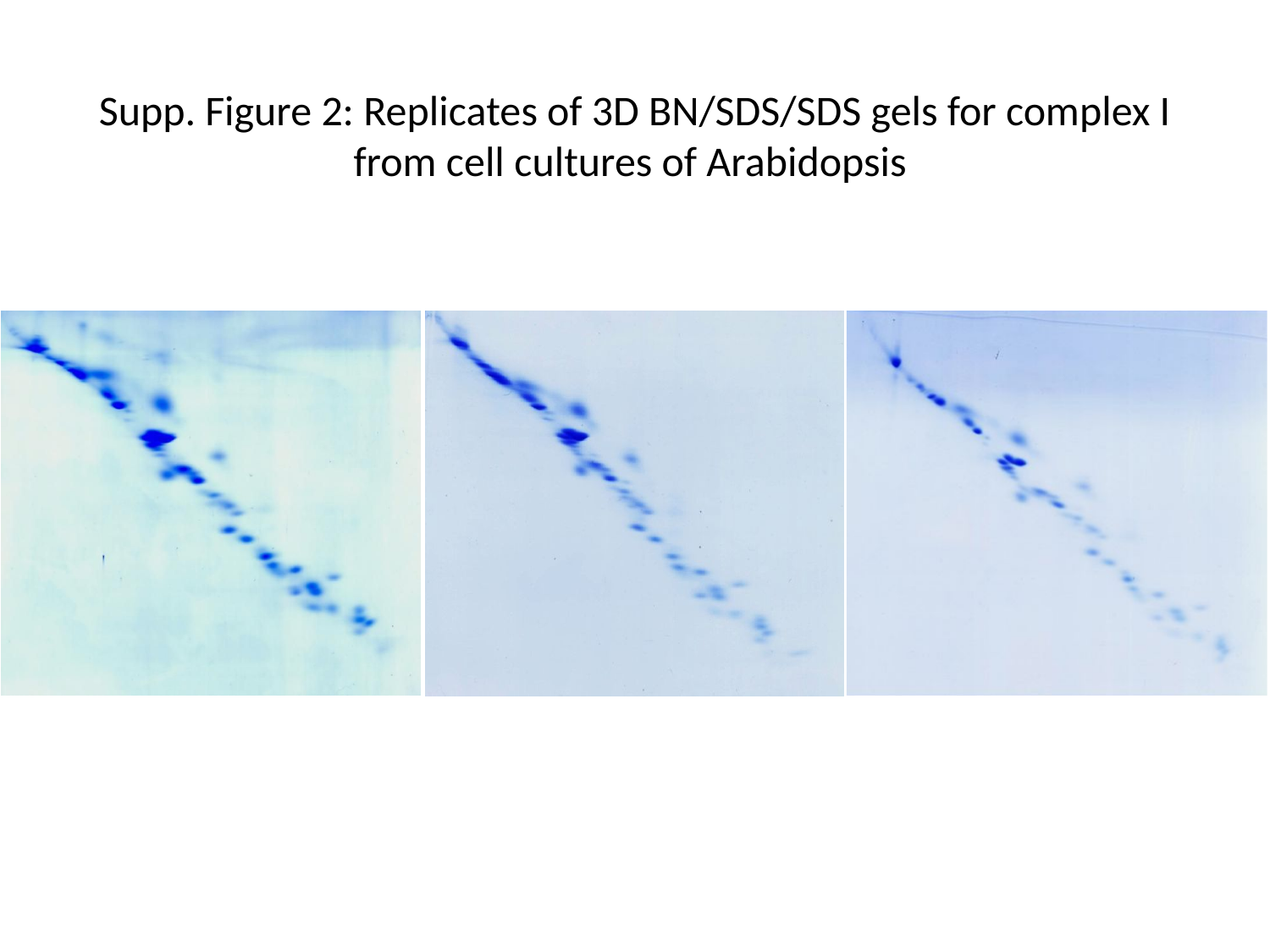

Supp. Figure 2: Replicates of 3D BN/SDS/SDS gels for complex I from cell cultures of Arabidopsis

## Slide 3
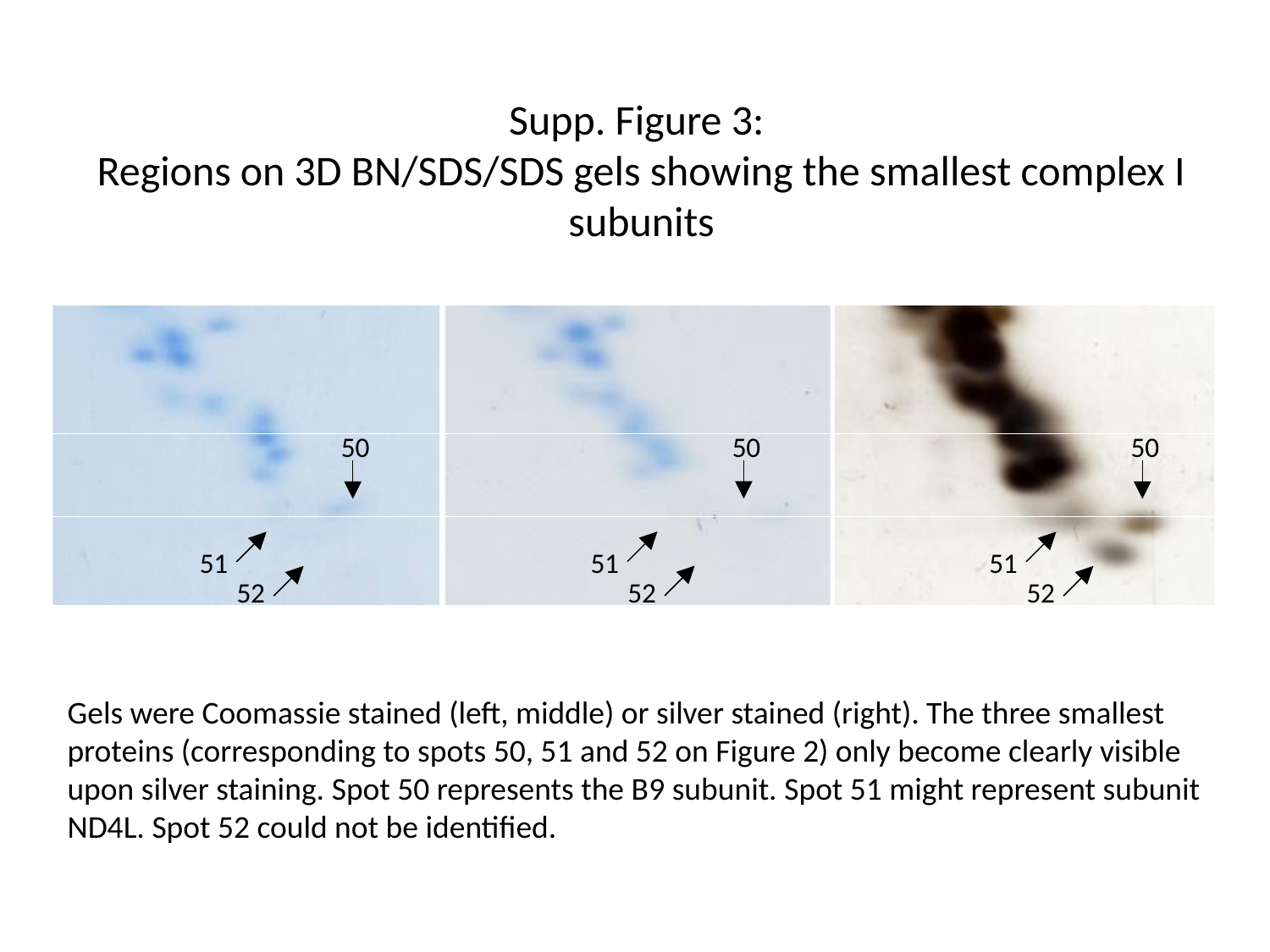

Supp. Figure 3:
Regions on 3D BN/SDS/SDS gels showing the smallest complex I subunits
50
50
50
51
51
51
52
52
52
Gels were Coomassie stained (left, middle) or silver stained (right). The three smallest proteins (corresponding to spots 50, 51 and 52 on Figure 2) only become clearly visible upon silver staining. Spot 50 represents the B9 subunit. Spot 51 might represent subunit ND4L. Spot 52 could not be identified.

## Slide 4
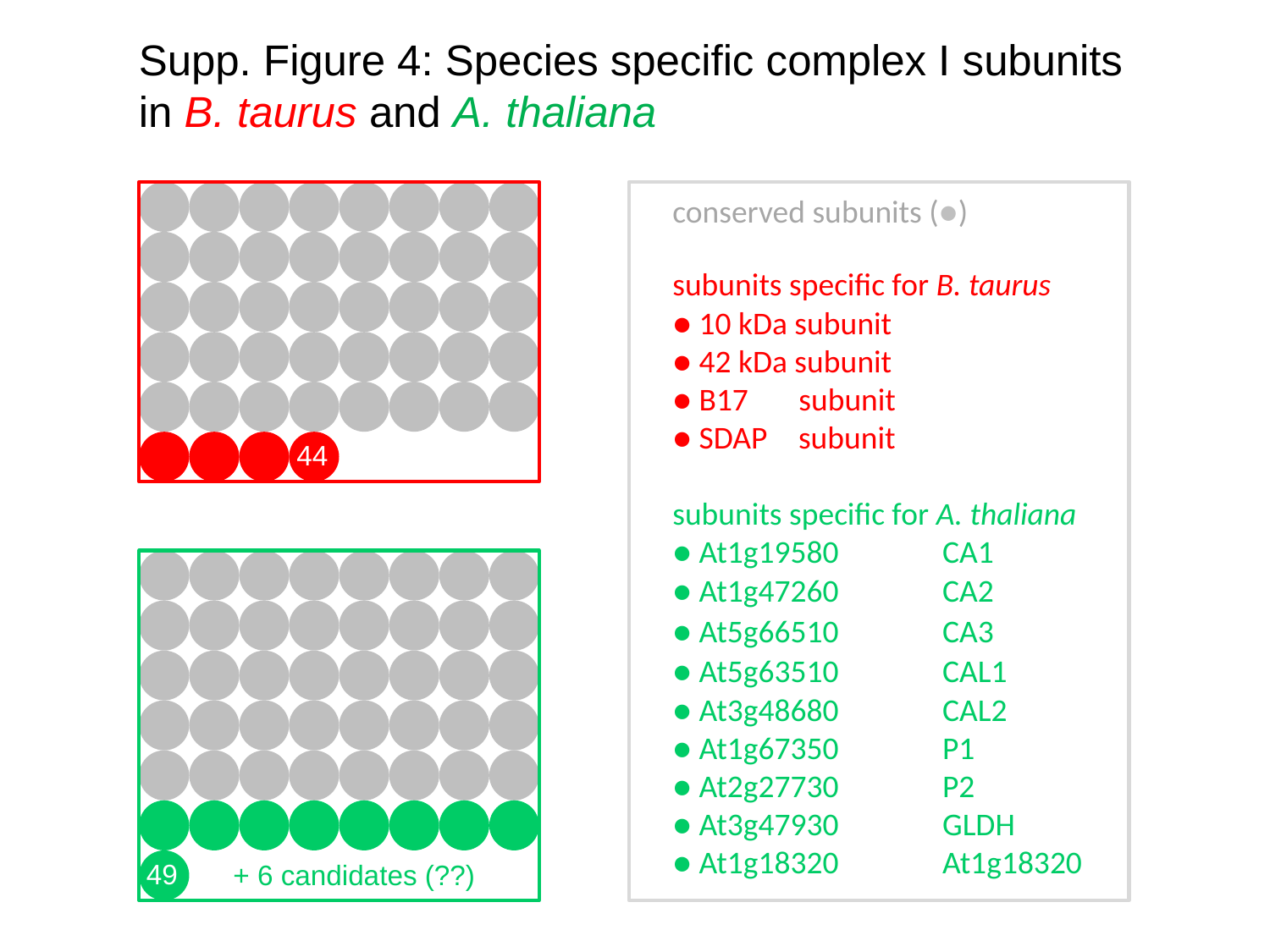

Supp. Figure 4: Species specific complex I subunits
in B. taurus and A. thaliana
| conserved subunits (●) | |
| --- | --- |
| subunits specific for B. taurus | |
| ● 10 kDa subunit | |
| ● 42 kDa subunit | |
| ● B17 subunit | |
| ● SDAP subunit | |
| | |
| subunits specific for A. thaliana | |
| ● At1g19580 | CA1 |
| ● At1g47260 | CA2 |
| ● At5g66510 | CA3 |
| ● At5g63510 | CAL1 |
| ● At3g48680 | CAL2 |
| ● At1g67350 | P1 |
| ● At2g27730 | P2 |
| ● At3g47930 | GLDH |
| ● At1g18320 | At1g18320 |
44
49
+ 6 candidates (??)

## Slide 5
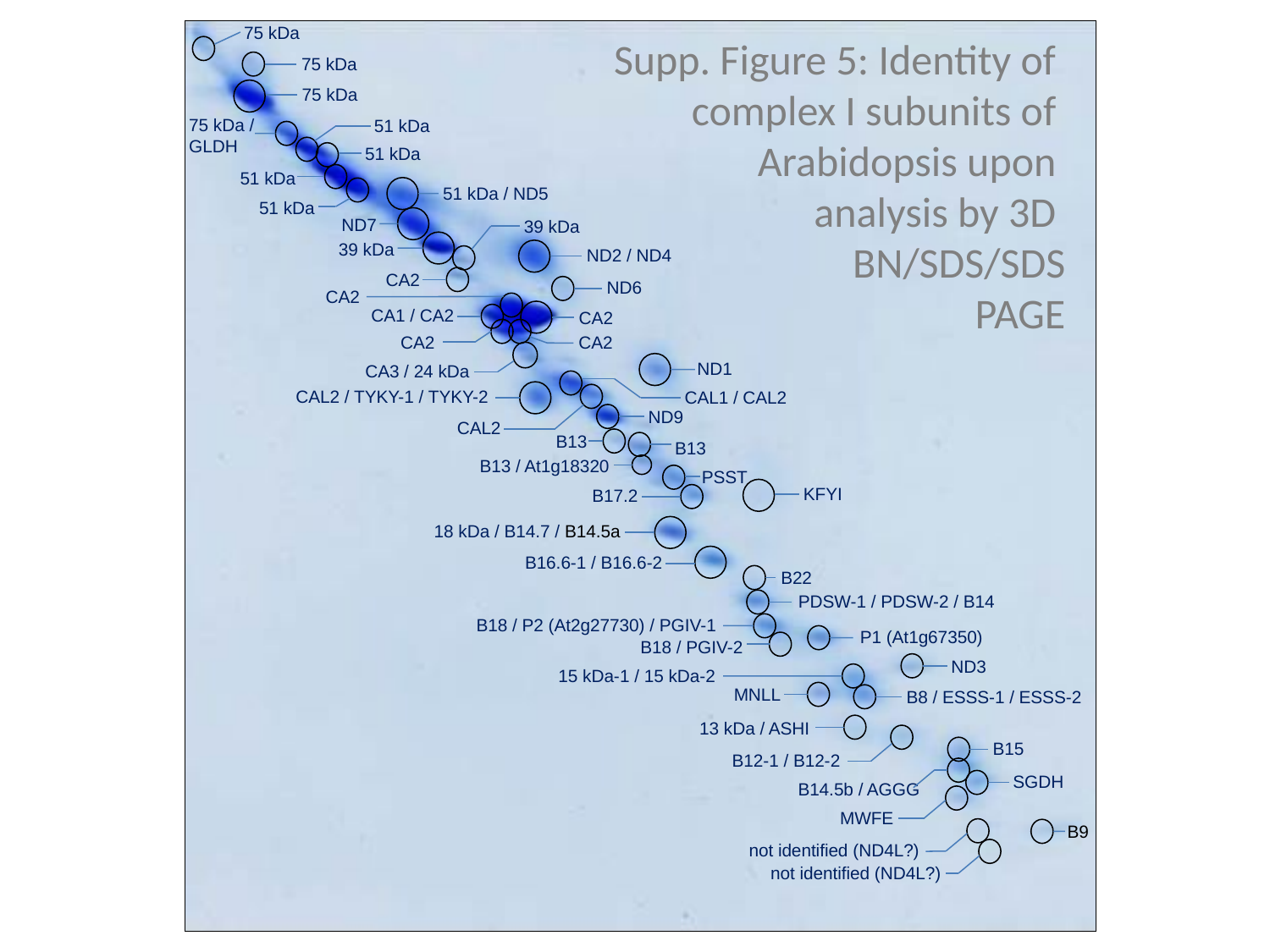

75 kDa
Supp. Figure 5: Identity of
complex I subunits of
Arabidopsis upon
analysis by 3D
BN/SDS/SDS
PAGE
75 kDa
75 kDa
75 kDa /
GLDH
51 kDa
51 kDa
51 kDa
51 kDa / ND5
51 kDa
ND7
39 kDa
39 kDa
ND2 / ND4
CA2
ND6
CA2
CA1 / CA2
CA2
CA2
CA2
ND1
CA3 / 24 kDa
CAL2 / TYKY-1 / TYKY-2
CAL1 / CAL2
ND9
CAL2
B13
B13
B13 / At1g18320
PSST
KFYI
B17.2
18 kDa / B14.7 / B14.5a
B16.6-1 / B16.6-2
B22
PDSW-1 / PDSW-2 / B14
B18 / P2 (At2g27730) / PGIV-1
P1 (At1g67350)
B18 / PGIV-2
ND3
15 kDa-1 / 15 kDa-2
MNLL
B8 / ESSS-1 / ESSS-2
13 kDa / ASHI
B15
B12-1 / B12-2
SGDH
B14.5b / AGGG
MWFE
B9
not identified (ND4L?)
not identified (ND4L?)
